# Supporting Police Well-Being Through an Adaptive Shift Management System: Co-Design Study

**DOI:** 10.2196/69986

**Published:** 2025-08-28

**Authors:** Olumuyiwa Temitope Ayorinde, Huseyin Dogan, Festus Fatai Adedoyin, Nan Jiang, Fiona Bitters, Sara Dempsey

**Affiliations:** 1 Department of Computing and Informatics Faculty of Science and Technology Bournemouth University Poole United Kingdom; 2 Hampshire Constabulary Hampshire and Isle of Wight United Kingdom

**Keywords:** shift work, police personnel, stress, health, police well-being, digital health, shift management

## Abstract

**Background:**

Police personnel work under challenging conditions commonly associated with complex shift patterns, unpredictable last-minute changes, and high stress levels, with shift work identified as the major contributor to police personnel health and well-being challenges. These challenges negatively impact their mental well-being, physical health, and job performance, leading to potential health concerns such as fatigue, poor sleep, long-term physical disabilities, anxiety, and poor work-life balance. Existing digital interventions fail to address the needs of shift workers due to focusing solely on conventional 9-to-5 schedules. This gap highlights the need for tailored interventions that incorporate shift management systems into health and well-being applications to support emergency service personnel.

**Objective:**

This study aimed to co-design a shift management system that can be incorporated into a well-being app tailored to address the health and well-being challenges caused by shift work in police personnel.

**Methods:**

This study used interactive management methodology combined with user-centered design and cocreation to facilitate 6 co-design workshops with a diverse group of stakeholders. Each session was structured around idea generation, structural prioritization, and iterative prototyping. User-centered design principles such as persona mapping, scenario walk-throughs, and structured feedback exercises were integrated into the workshop sessions to ensure that the system met diverse user needs. Data were analyzed through participatory feedback and thematic analysis, which allowed for continuous iteration and prioritization of system features based on stakeholder inputs.

**Results:**

Participants highlighted the need for a shift management system capable of managing complex and variable shift schedules with real-time adaptability and support for work-life balance. Thematic analysis revealed shift management challenges such as limited flexibility in accommodating schedule changes and issues managing rotating shift patterns. In response to these identified challenges, a prototype was developed that included features such as bulk creation and modification of shift schedules, shift customization, and visualization tools for monitoring and identifying shift trends and reusable patterns for efficiency. This study demonstrated that integrating a schedule management system into a well-being app could provide personalized support based on users’ shift schedules. The integration showed significant potential in supporting the health and well-being of police personnel.

**Conclusions:**

The co-designed shift management system demonstrated strong feasibility and high acceptability among the participants. The integration of shift scheduling into a well-being app can provide tailored support across several domains such as nutrition, hydration, sleep, and physical activity. This combination shows promise in providing a sustainable approach to enhancing the health, well-being, and work-life balance of personnel working in high-stress occupations such as policing.

## Introduction

### Background

Police work is widely recognized as one of the most stressful occupations, characterized by exposure to violence, trauma, death, heavy workloads, and irregular hours [1]. These stressors often lead to high rates of psychological stress, including anxiety and posttraumatic stress disorder [2]. High levels of stress among police officers have been linked to poor sleep quality, fatigue, malnutrition, long-term physical disabilities, sedentary lifestyles, impaired cognitive function, increased risk of cardiovascular disease, poor work-life balance, less family time, and mental health issues [3]. Police officers working afternoon and night shifts reportedly experience more stress due to the physically challenging situations and traumatic events they encounter, with some resorting to frequent alcohol consumption as a coping mechanism, which can lead to substance abuse issues [4,5]. Research has shown that UK police officers experience alcohol misuse at higher rates than their counterparts in continental Europe [5].

Sleep disorders are prevalent among police officers, with 54% of officers reporting issues, particularly those working night shifts [6]. Poor sleep quality and fatigue have been found to impair judgment, increasing the likelihood of mistakes that could endanger the public and the officers themselves [7]. The cumulative impact of these stressors is significant and often leads to lower job satisfaction and a higher likelihood of officers leaving the force among shift workers compared to non–shift workers [8].

Unlike conventional work environments, police officers operate within a highly disciplined structure, requiring officers to adhere to schedules that often involve last-minute changes, extended hours, or operational unpredictability. For these reasons, shift work increases the effect of occupational stressors in policing [1,6,9,10]. Research has suggested that shift workers face greater occupational stress and injury risk and reduced quality of life [11,12]. A well-being survey in partnership with the National Police Wellbeing Service in the United Kingdom highlights key challenges in the police workforce, including high workloads, emotional demands, and declining emotional energy at its lowest since 2020 [8]. These findings highlight the need for digital interventions that can address negative well-being effects of shift scheduling on police personnel. Digital solutions offer advantages such as real-time adaptability, interoperability, scalability, and cost-effectiveness, which makes them suitable for supporting personnel working under unpredictable and dynamic work schedules [13].

Digital technologies can be used to design solutions for health and well-being interventions that increase access to health information, promote healthy behavior, and improve quality of life [14]. Although digital technologies have increasingly been used to support physical, mental, and social health [15], current digital health and well-being applications often fail to meet the needs of police workers. Many existing mobile health (mHealth) apps are designed around standard, traditional 9-to-5 work schedules, making them unadaptable to professions characterized by last-minute changes, extended work hours, and certain schedule unpredictability [2]. Existing solutions often fail to account for the complex relationship among irregular shift schedules, frequent and unpredictable shift changes, and their profound impact on the overall health and well-being of these workers [2]. Buckingham et al [16] highlighted the frustration of police officers toward mHealth apps that do not cater to the realities of their working condition.

Given the unique pressures, high levels of occupational stress [12], sleep disturbances [6], and well-being risks [3,10] associated with shift work, there is a growing need for digital health interventions (DHIs) that not only accommodate operational demands but also promote long-term well-being. While many mHealth apps offer general stress or lifestyle support, few incorporate real-time shift data to personalize their recommendations, representing a critical gap in current digital health and well-being interventions.

This study sought to bridge this gap by cocreating a shift management system designed with input from police personnel that can be integrated into a health and well-being app. This system will support real-time adaptability and last-minute schedule changes and offer personalized and tailored well-being prompts based on police workers’ actual schedules. The integration of operational schedules and proactive support can significantly reduce occupational stress, improve mental health and physical well-being, and enhance job performance, ultimately contributing to public safety.

This study advocates for the role of a digital shift management system tailored to police personnel to reduce the health and well-being challenges associated with shift and irregular work schedules. It makes several innovative contributions: first, it highlights the well-being challenges associated with unique police work schedules; second, it demonstrates the application of the interactive management (IM) cocreation methodology in developing a digital shift management system; and, third, it presents a case for integrating a shift scheduling system with a mobile well-being app as a novel strategy to support real-time adaptability and health promotion.

### Literature Review

Police work is commonly viewed as a high-risk and stressful occupation associated with several stressors that negatively impact police personnel’s physical and mental health [1]. Police stressors can be divided into 3 categories: administrative and organizational pressures, physical and psychological threats, and a lack of internal and external support [12,17]. Administrative and organizational stressors include judicial demands, limited decision-making authority, public criticism, and work-family conflicts [1,17]. Physical and psychological threats arise from exposure to violence, death or injuries, high-speed chases, traumatic incidents, and night shifts [1,12]. Finally, strained relationships with colleagues and supervisors, inadequate departmental support, and political pressure contribute to the overall lack of support [12,18,19].

Research suggests that administrative stressors are reported more frequently than physical threats [20]. The 5 most common stressors experienced by police officers include managing family disputes, dealing with felonies, understaffing, quick decision-making, and working with underperforming colleagues. Stressors such as using force, witnessing the death of a colleague or a child, or being responsible for killing someone on duty are not common but are highly rated as they tend to cause profound psychological trauma [1,17,20]. Female officers report lack of support as their main stressor [20], whereas their male counterparts identify operational duties as the main source of their stress [6,20,21]. Purba and Demou [22] highlighted the impact of occupational stressors such as lack of support, working long hours, high work demand, and administrative and organizational pressure [23], which have been significantly associated with negative mental health outcomes among police personnel [21].

Occupational stress is increasingly recognized as a significant concern, with well-documented negative effects on employees’ health and wellbeing, including a heightened risk of cardiovascular diseases [24]. In the 2023 to 2024 financial year, >14,500 police officers across 43 forces in England, Wales, and Scotland were absent from work due to stress, depression, anxiety, or posttraumatic stress disorder [25]. Police forces in Dorset and Cleveland reported alarmingly high rates of work-related mental health absences, with 343% and 526% increases, respectively [25].

Cartwright and Roach [26] reported that work absence due to mental health issues among officers nearly doubled to approximately 8.82% in the last 10 years [27]. Worryingly, 39% of those who took mental health absent leave subsequently required an additional period of absence, with the primary causes of these absences being stress, trauma, and psychological health issues. Gender differences were observed, with 75% of female officers reporting worsened mental health challenges due to their work compared to their male counterparts [26]. According to the Police Federation of England and Wales [28], 43% of officers reported finding their job extremely stressful, whereas 82% reported having experienced stress, anxiety, or several challenges with their health and well-being. High workload (60%), poor work-life balance (52%), and working in shift patterns (38%) were identified as the top 3 contributors to work-related health and well-being challenges reported by police staff [29].

While general occupational stressors are prevalent, shift work, particularly night shifts, has been identified as the leading contributor to negative physical and mental challenges among emergency services such as the police force [10,12,17]. Policing commonly involves day or night working time occurring in a fixed, rotating, or irregular shift schedule. Police workers with long and irregular shift patterns are at higher risk of burnout and emotional exhaustion than those with fixed shift schedules [10,30].

According to the 2023 National Police Wellbeing Survey, 43.5% of police officers working shifts reported experiencing insufficient sleep compared to 32.7% of non–shift workers. Subsequently, 25.5% of shift-working officers reported experiencing disturbed sleep versus 22.4% of non–shift workers [8]. Similarly, police staff working shifts also reported high rates of disturbed sleep (24.2%) and insufficient sleep (38.8%) compared to their non–shift-working counterparts with 16.4% and 24.8%, respectively [8]. This aligns with the findings of Fekedulegn et al [31], who reported that 54% of police officers experienced poor sleep quality, with a rate of 69% among night shift workers [6,31]. A longitudinal study involving 464 officers found that night shift workers took sick leave at the rate of 4.37 sick days per 10,000 working hours, which is nearly 3 times the sick leave rate of day shift workers, with 1.55 sick days per 10,000 working hours. The risk of absence due to sickness was 182% higher for night shift officers and 129% higher among officers who were overweight [31].

Extended absences of ≥3 days were 65% more common for night shift workers and 50% more common for afternoon shift officers than for their day shift worker counterparts [31]. These statistics show the accumulating physical toll associated with shift work, particularly when combined with occupational stress [4,6,12,17,31]. Operational work culture further amplifies these issues. The Police Federation Pay and Morale Survey 2022 report found that 33% of officers felt pressured to work long hours, and 47% reported working >48 hours per week [29]. Alarmingly, 38% of officers were unable to take their full annual leave entitlement, and 61% had ≥2 rest days canceled [29]. The practice of using annual leave to recover from illness was reported by 33% (physical ill-health) and 42% (psychological ill-health) of workers, whereas 67% admitted to attending work while unwell [29]. According to the 2023 Pay and Morale Survey report, 1 in 5 officers experienced >10 roster changes in a single year, largely due to unpredictable demands [29]. These frequent changes contribute to mental strain and work-life disruption, further compounding their stress.

Key insights from research on shift scheduling has shown that police officers prefer rapidly forward-rotating shift systems with at least 16 hours of rest between shifts despite these schedules offering fewer days off than compressed systems [32]. This preference highlights the importance of shorter consecutive workdays, reduced weekly hours, and adequate rest periods in promoting the health and well-being of officers [32]. These findings challenge claims that shift workers prefer compressed systems with more days off [33,34], highlighting the diversity of individual preferences. Age, current work hours, and previous experiences with shift schedules significantly influence these preferences [34]. Compared with officers with regular shift schedules, those working shifts experience high levels of fatigue, work overload, and reduced task control, resulting in lower job satisfaction and a higher likelihood of leaving the force [8,17].

These widely varied preferences highlight the need for tailored development of digital shift management systems that can address the mental health challenges associated with shift work, offering more personalized and effective solutions [32,35]. Despite the growing use of DHIs to address health and lifestyle challenges, many digital interventions are not tailored to the needs of shift workers [36]. Due to the continually changing shift pattern in policing, the effectiveness of these digital interventions is often limited in mitigating work-related stress, depression, and anxiety among emergency service personnel [37].

A study exploring the views of UK police personnel identified a significant shortcoming in existing digital health and well-being interventions, which is their lack of personalization for shift-based working patterns. These interventions are often misaligned with the operational realities of police work, particularly due to their limited adaptability to variable and irregular shift schedules [3]. For example, the Headspace mindfulness app [37] provides mindfulness content but does not adapt it to users’ shift patterns. Similarly, the Sleepfit app [38], which focuses on sleep health, lacks real-time integration with varying shift schedules. The Healthier Outcomes at Work app [39] addresses workplace stress management but fails to consider the unique demands of shift work. The Healthy Minds Innovation app [40] provides mindfulness and meditation support but does not integrate the intervention with the users’ work schedules. A similar scenario occurs with the #SWPMoveMore Challenge intervention [41], which encourages physical activity but fails to incorporate shift schedules into its data tracking and recommendations. Although these interventions target different aspects of health and well-being, their lack of shift-specific personalization limits their effectiveness for the target population. This represents a key limitation for populations such as police officers and emergency workers with unpredictable and irregular work patterns.

Research on improving shift scheduling and fatigue management among police workers highlights several recommendations to ensure health, safety, and performance [42], such as involving employees in scheduling decisions, maintaining regular work hours, and ensuring that any shift changes rotate forward rather than backward [11,42]. These recommendations aim to reduce the adverse effects of shift work on both the physical and mental well-being of police workers. By implementing these recommendations, police workers and other workers in similar high-stress environments can benefit from effective and sustainable shift scheduling systems that enhance their overall well-being and performance [12,30,42].

mHealth apps can play a crucial in addressing mental health and stress‑related concerns among emergency service personnel [43]. In a public engagement study conducted by the National Health Service England, the stakeholders emphasized that effective DHIs must be engaging, accessible, and tailored to users’ needs, with features such as gamification, rewards, personalization, and responsive feedback channels identified as crucial for sustainable user engagement and satisfaction [43,44]. Despite the increasing use of health and well-being apps, many digital interventions fail to address the unique needs of shift‑working officers. Mental health issues such as stress, depression, and anxiety remain prevalent due to existing apps often suffering from low adherence, insufficient customization for irregular schedules, and weak user engagement due to failure in accommodating fluctuating work patterns [45,46].

Marston et al [47] proposed a collaborative approach for developing DHIs specifically for emergency services by bringing together researchers, emergency staff, stakeholders, designers, and developers. They recommended not only establishing a framework for developing and implementing DHIs but also ensuring interoperability and consistent use of these apps across different emergency service organizations [47,48]. There is evidence suggesting that personalized, on‑demand digital tools frequently outperform physical therapy sessions in managing work‑related stress [49], yet many current DHIs lack the behavioral tailoring to users’ routines, adaptative feedback responses to real-time user data, and shift‑specific features such as rota or schedule changes necessary to meet the evolving needs of emergency personnel.

Kerr et al [50] identified key concerns among employees using DHIs for managing stress—health impact, privacy, autonomy, identity, and accountability could further hinder the adoption of DHIs [3,48]. While most of the employees in the aforementioned study were open to using digital stress management tools, they strongly preferred options that integrated seamlessly into their daily routines and prioritized data privacy. Weerasekara and Smedberg [2] found that users preferred stress management interventions that involved self-help modules, personalized activities, and peer support [51]. Howe et al [49] suggested developing digital interventions that reduce work-related stress, such as by integrating a variety of stress relief content, including personalization for users, and introducing stress-reducing strategies such as longer break periods [49]. Tailoring these interventions to individuals’ experiences, needs, and preferences was identified as extremely essential for engagement. Building on these preferences, participants in previous studies have suggested integrating peer support; expert guidance; and gamification features such as social recognition, badges, mini games, and group challenges to boost user adherence and engagement [44,52]. Finally, participants have emphasized that privacy, security, and confidentiality are essential requirements for digital interventions [3,50,53].

To bridge these gaps, future digital health and well-being interventions for shift workers should be developed using inclusive and user-centered approaches that involve diverse end users [44]. They should also integrate adaptive shift management features that help users plan their activities, nutrition, exercise, sleep, and recovery around irregular schedules, thereby promoting a sustainable work-life balance [32,42]. Adherence to ethical design principles; transparent data privacy practices; and maintenance of active partnerships among emergency services, researchers, and developers will allow for iterative refinement and robust evaluation of developed digital health and well-being interventions [54]. The next step is to organize cocreation workshops with serving police staff and other important stakeholders to gather detailed, shift‑specific requirements, thereby ensuring the development of future DHIs reflect the real‑world demands of shift work.

## Methods

### Research Design

#### Overview

This study used a qualitative research approach using a series of IM workshops to gather insights on the scheduling needs and preferences of police workers. This iterative, participatory method enabled in-depth exploration of the participants’ experiences with existing shift management systems and how they affect their well-being. This approach enabled the researchers to probe participants for responses, encourage collaborative discussion, and uncover the challenges associated with shift-based police work. Thematic analysis (TA), nominal technique, and interpretive structural modeling were used to analyze and structure the data collected.

#### Population and Sample

Participants were selected using a combination of purposeful and convenience sampling. Individuals directly involved in shift pattern coordination, operational management, frontline policing, and digital system design were targeted. This approach ensured that the perspectives of those most involved in implementing shift scheduling systems were represented. The participants were affiliated with a regional police force in the southern region of the United Kingdom. The sample comprised a chief superintendent of police, a police operations manager, an expert in human-computer interaction (HCI), a digital health expert, an academic researcher, and a shift management administrator who manages existing shift scheduling systems. This group represented a cross-section of technical, operational, and strategic expertise necessary for effective system co-design.

Purposive sampling ensured the inclusion of participants with specialist knowledge, whereas convenience sampling accommodated practical constraints related to availability [55,56]. Although broader representation was limited by logistical and ethical constraints, the selected participants were well suited to the aims of this study, representing a variety of roles; levels of responsibility; and those who could provide meaningful, experience-based feedback on system requirements and implementation feasibility. All participants were invited to join interactive co-design workshops depending on availability and relevance to specific development stages. Meetings were held in person or via web-based platforms (eg, via Microsoft Teams) depending on participant availability and logistical considerations.

#### Instruments

The primary instrument used for data collection was a semistructured discussion guide iteratively developed to support each of the 6 co-design workshops conducted. Early sessions focused on requirement gathering, terminology refinement, and system expectations, whereas later sessions addressed feature testing; interface usability; and refinement of core components such as bulk shift creation, interactive visualization, and preconfigured shift patterns (eg, gold and silver shift pattern template).

Five of the 6 meetings were conducted virtually via Microsoft Teams, recorded using the platform’s built-in functionality, and transcribed using Microsoft Teams’ automatic transcription feature. The session with the shift schedule meeting (meeting 3) was held in person and was recorded using a mobile recording device and manually transcribed by the research team.

The discussion guide was iteratively refined to reflect the evolving objectives of the project. It enabled (1) open-ended exploration of contextual challenges such as inflexible scheduling and well-being concerns; (2) targeted feedback on design elements, including shift visualization tools, terminology, and shift rota flexibility; and (3) prioritization of feature requirements through structured prompts and facilitated consensus-building exercises.

#### Trustworthiness

To ensure the trustworthiness of the qualitative process and findings [55], several strategies were used: (1) pilot-testing of the IM method with internal stakeholders to refine the guide and facilitation approach; (2) member checking during workshops, including real-time clarification and postsession return of professionally transcribed data to key participants for validation; and (3) triangulation of participant feedback across multiple sessions and user groups to identify consistent patterns and diverse perspectives.

#### Structure of the Co-Design Process

This study adopted a cocreation and user-centered design (UCD) approach structured around six 60-minute IM-facilitated workshops. The workshops integrated UCD principles such as persona mapping, scenario walk-throughs, and structured feedback exercises.

Meetings 1, 2, 4, 5, and 6 involved a core group of stakeholders, including a chief superintendent of police, a police operations manager, an expert in HCI, a digital health expert, and an academic researcher. These sessions focused on initial scoping, terminology development, interface functionality, technical feasibility, contextual constraints, user workflow, feature walk-throughs, usability feedback, and validation of the system.

Meeting 3 involved the operational shift scheduling unit responsible for coordinating shift patterns across departments. This involved demonstration of how the existing police scheduling system operates and provided a deep dive into management of complex shift patterns, the combination of multiple shift types, and the prepopulation of base shift pattern templates (eg, gold or silver shift patterns) across different policing departments.

Meeting 6 served as a final validation session focusing on implementation readiness and final design insights. This was followed by nominal group techniques and interpretive structural modeling. This process supported the prioritization of user-identified needs and clarified dependencies between proposed features.

### IM Session

#### Overview

IM is a methodology that facilitates collaborative decision-making to tackle complex problems [57]. IM promotes communication and shared understanding among groups of stakeholders or participants to develop a deep understanding of a problem, explore meaningful solutions, and collectively agree on a defined course of action to solve the problem [58]. Unlike traditional focus groups, IM involves the identification of domain experts, consensus building, iterative idea development, and prioritization of the solution [59]. It is especially effective in solving problems associated with high complexity, diverse stakeholder viewpoints, and evolving constraints, making it well suited for addressing the multifaceted challenges surrounding police shift management and system design.

The phases of IM are outlined in the following sections.

#### Planning Phase

The planning stage involved collaborative work with key stakeholders to develop a shared understanding of the current system and the problem space. Activities included defining the scope, identifying affected actors, and cocreating guiding questions for the workshops. This ensured that workshop content addressed real-world operational challenges such as inflexible scheduling, system usability, and well-being implications [58].

#### Workshop Phase

The workshop sessions were structured around the IM framework’s 3 core dimensions: context, content, and process. Each session began with a contextual overview provided by the facilitator to ground participants in the session’s objectives and revisit insights from previous sessions [58]. This approach ensured continuity and a cumulative design process. Participants were then guided through idea writing exercises, each triggered by carefully framed, open-ended questions relevant to the project’s aims.

#### Idea Writing

Participants exchanged ideas, refined them through discussion, and engaged in nominal group technique sessions. Participants collaboratively edited and clarified problem statements and then assigned priority ratings to the ideas, helping the group identify the most critical needs. The top-rated ideas were transformed into design objectives and used to construct interpretive structural models [59]. This modeling process helped identify interdependencies between ideas, clarifying design priorities and informing system architecture decisions.

#### Follow-Up Phase

Insights generated during each session were synthesized into actionable requirements and integrated into subsequent design iterations. Although no separate follow-up interviews were conducted, participant feedback was captured in the sessions and validated through ongoing collaborative engagement [58]. When new challenges emerged, such as departmental shift variation or ambiguous terminology, they were integrated into planning for the next workshop, maintaining the recursive and adaptive nature of the IM methodology.

### Cocreation Process

Cocreation has been linked to several methods, such as co-design and coproduction, as well as design approaches such as design thinking. Cocreation is suggested to be also known as participatory design [60] and has been used to develop several digital health care interventions. Early involvement of users and other major key stakeholders in the design, development, and evaluation of digital health solutions has proven to be an important factor in the success of these interventions [61]. Interventions developed using cocreation have been reported to result in improved health outcomes [62]; improved mental health [63]; enhanced quality of life [64]; and enhanced overall well-being consisting of social, psychological, and physical aspects [65]. Research has shown that the potential actors involved in a cocreation process are the end users, stakeholders (mostly referred to as the nonacademic stakeholders), and academic researchers. The end users are the population that the intervention is targeted to, and the stakeholders are groups that are concerned with the implementation of the interventions, whereas the academic researchers conduct the research [65]. Involving end users in the cocreation of interventions has been reported to increase satisfaction, use, and effectiveness of the intervention due to the end user participation in creating solutions targeted to their problems and needs [66,67].

### UCD Approach

UCD is an approach for developing interventions that involves and prioritizes the end users in every stage of the solution development phase. The involvement of users in every step of the development phase ensures high quality and user acceptance of the developed interventions [68]. UCD involves the iterative process of understanding the user needs and requirements through research, designing a prototype to obtain user feedback, refining the design based on user feedback, testing and evaluating the design, and monitoring the intervention after launching [69]. Recently, UCD has been used to develop health-related interventions targeted at different health issues, such as pain management, health and well-being, and mental health [70].

**Figure 1:**
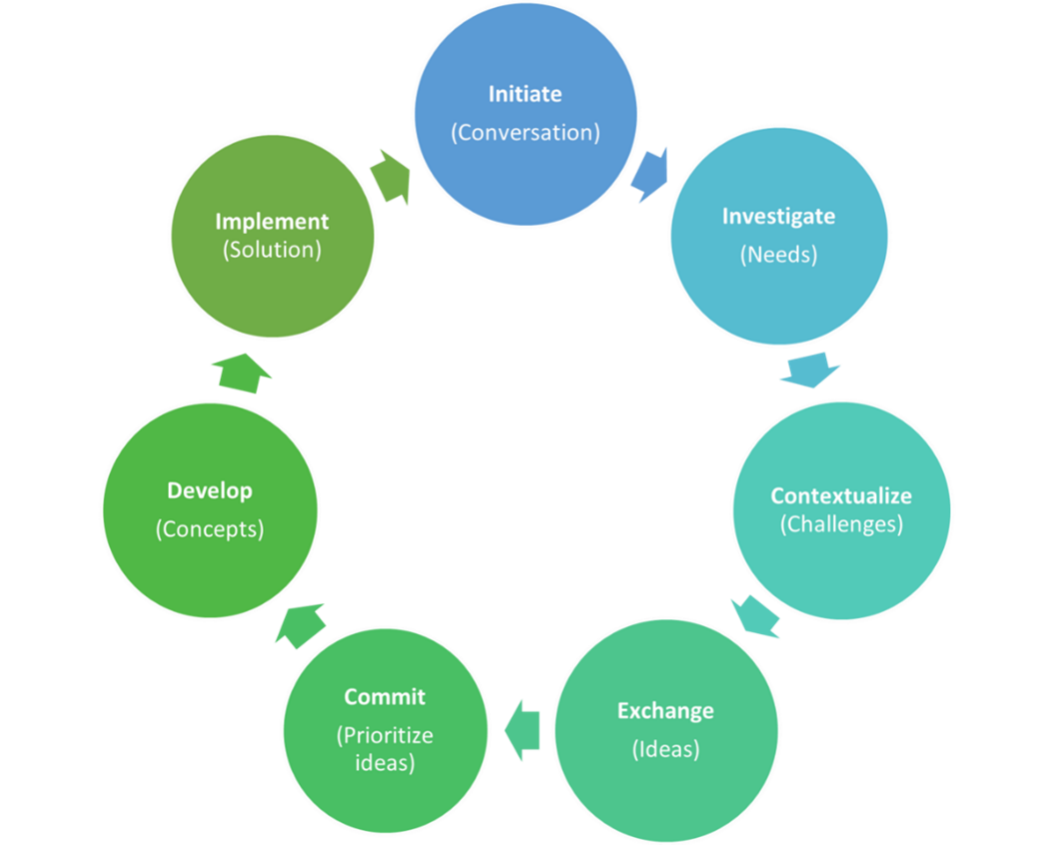
Cocreation process with embedded user-centered design principles.

This research combines innovative and design approaches in developing the shift management system (Figure 1 [71]) by exploring UCD techniques in the design phase of the cocreation process. This cocreated intervention was collaboratively developed and targeted at police workers to address stress arising from shift work.

### TA Process

TA was used to identify, analyze, and interpret patterns known as themes in qualitative data. TA uses data collected from participants’ perspectives, views, behavior, and experiences to identify patterns or themes regarding how the participants feel and think [72]. TA can be used to analyze datasets involving few participants and datasets from a large number of participants [73]. TA has proven to be very efficient in analyzing a wide range of data collected through qualitative techniques such as workshops, focus groups, observations, interviews, and story completion [72,74].

Before conducting the coding process for the TA, the meeting minutes were transcribed to ensure an accurate representation of the agreed discussions. After transcription, keywords were identified from the data, and codes were developed from these keywords, which were subsequently grouped into themes. To ensure a thorough understanding of the qualitative data, the transcribed documents were read multiple times, allowing the research team to gain familiarity with the content and context of the discussions [74]. This iterative reading process helped refine the coding process and ensured that the themes accurately reflected the core insights from the cocreation sessions (see Multimedia Appendix 1 for more details).

The process led to the identification of 57 codes in the initial coding process, which were further refined to 15 unique codes after merging similar codes to remove duplications. These codes were derived from structured trigger questions posed during the cocreation sessions (Table 1).

**Figure 2:**
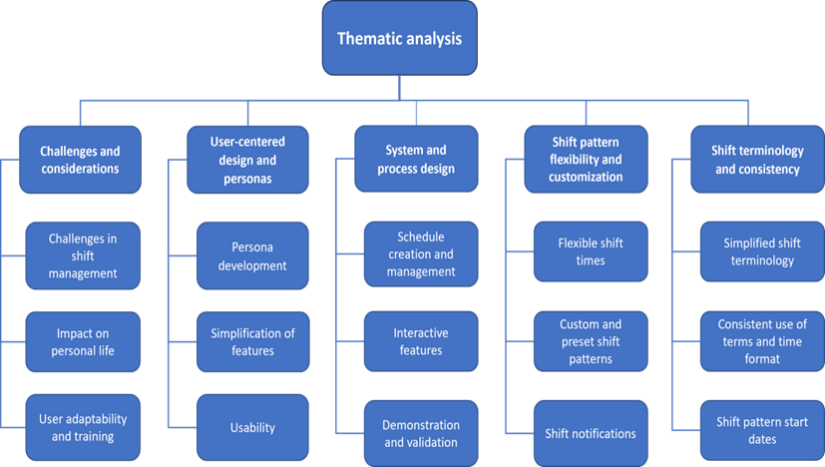
Thematic map of core themes from the interactive management–facilitated workshops.

The identified codes captured trends and patterns in the data, and after thorough synthesis, 5 overarching themes were identified (Figure 2) [70].

**Table 1:** Trigger questions and codes for creating themes from the cocreation sessions.

Trigger question	Codes for creating themes from participant responses
What are the main factors contributing to stress and well-being challenges caused by shift work and shift management?	Lack of shift flexibility to reduce stress, shift work–related stress, lack of support for work-life balance, issues managing rotating shifts, inconsistent shift scheduling, inadequate and inflexible shift pattern creation system for large teams (eg, 2000 users), disruption of personal life due to shift work, and varying shift start and finish times.
How should the system be designed to accommodate diverse user needs while ensuring ease of use?	Develop several detailed personas representing different user roles with unique schedules. These personas are used to guide design decisions to ensure that the functionalities meet the tailored needs of the user group. One proposed approach involves using the cyclelike approach of creating and managing shift patterns while ensuring easy navigation for users. The interface should be interactive and responsive while also reducing the number of steps required to complete a task.
Which key features are essential for efficient and effective shift schedule management?	Bulk creation of shift schedules; flexible modification of schedules; shift type monitoring through visualization; ease of creation and management of complex shift patterns; ability to merge rotating schedules; identification of shift trends and distribution through visualization; and simplified interactive process of creating, editing, and managing shift patterns.
What enhancements can be made to improve the flexibility and customization of shift patterns to better meet scheduling needs?	Daily shift time customization to accommodate varying personal and professional obligations, manual editing of individual days within a shift pattern for quick adjustments, creation of custom shift patterns with multiple finishing times within a single pattern, creation of shift patterns from existing base patterns, setup of recurring shift patterns with flexible cycles for users with regular nonidentical weekly or monthly schedules, and notification to alert users of upcoming shifts.
How can consistency and appropriate terminology enhance the understanding of the shift scheduling system?	Replacing shift terms with more understandable and familiar vocabulary, implementing consistent terminology to reduce confusion and improve communication, ensuring that shift patterns begin on a Monday to align with the traditional workweek, and use of the 24-h time format across the entire system to enhance clarity.

### Ethical Considerations

This study received ethics approval from Bournemouth University Research Ethics Committee (approval 58206). All participants, including those authors who took part in the co-design sessions, were provided with a participant information sheet. This document outlined the study’s purpose, procedures, data handling practices, and confidentiality provisions and the participants’ rights, including voluntary participation and the ability to withdraw at any time without consequence. Informed consent was obtained in writing from all participants after they were provided with a detailed participant information sheet. Following the ethical standards of the university, all participant data were anonymized during transcription and securely stored on password-protected and encrypted devices. Anonymized data may be made available for future ethically approved research in compliance with the ethical agreement, including use in presentations, reports, or publications.

## Results

### What Are the Main Factors Contributing to Stress and Well-Being Challenges Caused by Shift Work and Shift Management?

Participants were asked about the main factors contributing to stress and well-being challenges caused by shift work and shift management. The findings revealed several key stressors: a lack of shift flexibility to reduce stress, stress directly related to shift work, insufficient support for achieving a work-life balance, difficulties in managing rotating shifts, and inconsistent shift scheduling. In addition, participants highlighted the inadequacy and inflexibility of shift pattern creation systems, particularly for large teams (eg, 2000 users), along with the disruption of personal life due to shift work and varying shift start and finish times. These factors collectively contribute to stress and well-being challenges among shift workers (Table 2). A few quotes from the sessions include the following (*M* indicates “meeting” and *P* indicates “participant”):

Rotating shifts are difficult to manage, especially when we have to combine multiple patterns.

M3P1

Poorly designed shift patterns harm personal life and work performance.

M1P5

Flexible or predictable scheduling system would help maintain a better work-life balance.

M1P4

Struggle with the complexity of software made for large teams, especially when everyone has different schedules.

M1P2

**Table 2:** Identified stressors associated with shift work among police personnel.

Theme	Description	Quotes	Percentage
Scheduling constraints and fatigue risks	Lack of adequate rest and recovery time due to an irregular shift structure	“Current system has limited flexibility and inconsistent scheduling.” [M1P3] “I would appreciate reminders to take breaks when I’ve been doing back-to-back shifts.” [M5P5] “The system should suggest breaks when we’ve worked several heavy shifts.” [M6P5] “The system should be able to warn me if I’m scheduled to work too many night shifts in a row.” [M6P4]	30.8
Insufficient support for work-life balance	Disruption of personal routines, relationships, and mental recovery caused by unpredictable shift schedules	“Flexible or predictable scheduling system would help maintain a better work-life balance.” [M1P4] “Poorly designed shift patterns harm personal life and work performance.” [M1P5]	15.4
Difficulty managing rotating shifts	Challenges in managing and combining multiple rotating shift patterns	“Rotating shifts are difficult to manage, especially when we have to combine multiple patterns.” [M3P1] “We should simplify things by using cycles instead of overly complex shift patterns.” [M4P1]	15.4
Incompatibility with real-world shift demands	Scheduling systems fail to accommodate diverse shift end times or departmental needs	“We have officers with varying finish times, one pattern doesn’t fit all.” [M4P3] “It’s hard to understand systems designed to manage shifts for thousands of staff with varying start and end times.” [M1P1]	15.4
Lack of clarity and an ineffective scheduling support tool	Stress caused by unclear guidance and a lack of user-friendly scheduling tools	“There should be a simple way to describe different shift options and how we use them.” [M6P1] “The system should give me clear instructions, like how to start a pattern on a certain day and repeat it.” [M6P2] “Users will benefit from being able to see trends that show recurring scheduling issues.” [M4P5]	23.1

### How Should the System Be Designed to Accommodate Diverse User Needs While Ensuring Ease of Use?

Participants were asked how the system should be designed to accommodate diverse user needs while ensuring ease of use. The findings suggest that the system should incorporate several detailed personas representing different user roles with unique schedules. These personas are intended to guide design decisions, ensuring that the system’s functionalities meet the tailored needs of various user groups. In addition, the design should follow a cyclelike approach for creating and managing shift patterns, ensuring easy navigation for users. The interface should also be interactive and responsive and minimize the number of steps required to complete tasks, facilitating a more efficient and user-friendly experience. A few quotes from the sessions include the following:

I think it would help to have a model or table that links user personas with shift patterns.

M2P1

We should create 5 to 10 fictional personas based on demographic data and real shift schedules.

M2P5

I would like to see a list of example characters with typical shift patterns to guide system use.

M2P3

We should simplify things by using cycles instead of overly complex shift patterns.

M4P1

**Figure 3:**
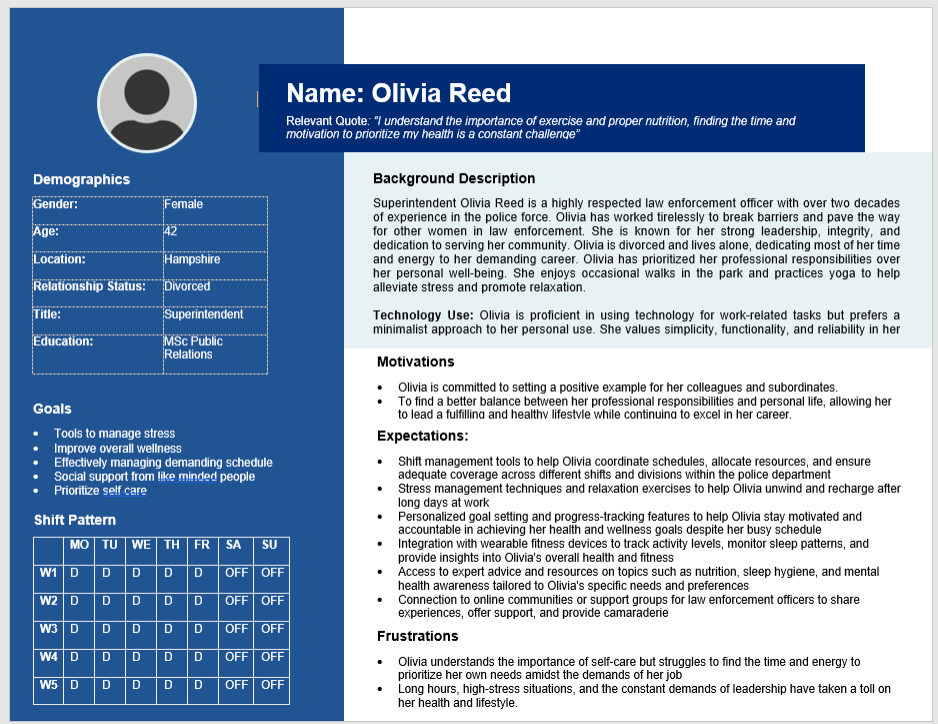
Sample persona showing stakeholders’ needs, motivations, and frustrations.

Personas have been reported to improve the identification of user needs and enhance quicker solution development [75]. Personas were used during the design phase to ensure that the proposed digital intervention met the needs of the diverse user groups (Figure 3).

### Which Key Features Are Essential for Efficient and Effective Shift Schedule Management?

Participants were asked to identify the key features essential for efficient and effective shift schedule management. The findings highlight several critical features, including the ability to create shift schedules in bulk and modify them flexibly. Monitoring shift types through visualization, simplifying the creation and management of complex shift patterns, and merging rotating schedules were also noted as important. In addition, the ability to identify shift trends and distributions via visualization, along with a simplified and interactive process for creating, editing, and managing shift patterns, were deemed essential to optimizing shift management. A few quotes from the sessions include the following:

Users should be able to set different shift times for different days within the same pattern.

M4P4

I want to input my entire schedule in one go like a bulk pattern, not break it into separate parts.

M5P2

It would be nice to merge two patterns together and use them as one ongoing schedule.

M5P2

Interactive visual tools would make it easier for me to track progress over time.

M4P1

The key features in Table 3 were derived using nominal group techniques (Table 4). Five participants independently ranked each proposed feature on a scale of 1 (least important) to 5 (most important). The scores were then summed to determine relative importance.

**Table 3:** Key ideas generated during the cocreation sessions.

Idea number	Idea description
1	Bulk creation of shift schedules
2	Flexible modification of shift schedules
3	Shift type monitoring through visualization
4	Ability to merge rotating schedules
5	Daily shift time customization to accommodate varying personal and professional obligations
6	Possibility for shift patterns to contain a combination of several shift types
7	Manual editing of individual days within a shift pattern for quick adjustments
8	Creation and management of complex shift patterns
9	Setup of recurring shift patterns with flexible cycles
10	Notification to alert users of upcoming shifts
11	Replacing shift terms with more understandable and familiar vocabulary
12	Ensuring that the shift pattern begins on a Monday to align with the traditional workweek
13	Use of the 24-h time format across the entire system to enhance clarity
14	Creation of a schedule with multiple finishing times within a single shift pattern
15	Prepopulation of the base shift pattern
16	Ability of the created shift pattern to be reused and modified to create a new shift pattern

**Table 4:** Participant prioritization scores for user-generated ideas.

Idea number	Participant 1 score (1-5)	Participant 2 score (1-5)	Participant 3 score (1-5)	Participant 4 score (1-5)	Participant 5 score (1-5)	Total score
1	4	4	4	2	4	18
2	3	3	2	3	4	15
3	2	2	2	3	3	12
4	3	2	2	3	3	13
5	4	3	2	3	4	16
6	5	4	3	2	4	18
7	4	5	2	2	4	17
8	4	1	3	4	3	15
9	4	3	3	3	4	17
10	5	1	1	5	2	14
11	4	5	1	1	1	12
12	1	5	3	2	2	13
13	1	5	1	3	2	12
14	3	2	1	4	2	12
15	3	3	2	4	3	15
16	3	4	4	2	4	17

### What Enhancements Can Be Made to Improve the Flexibility and Customization of Shift Patterns to Better Meet Scheduling Needs?

**Figure 4:**
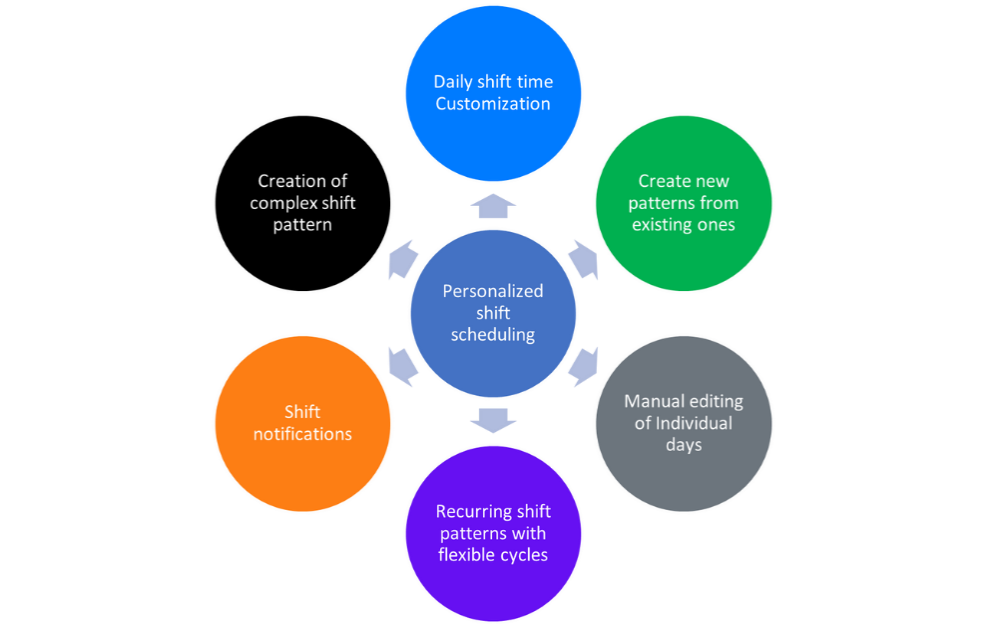
Customization and flexibility requirements in shift scheduling.

Participants were asked about the potential enhancements that can improve flexibility and customization of shift patterns to better meet scheduling needs. The findings indicated that daily shift time customization, allowing for adjustments to accommodate varying personal and professional obligations, is essential. Manual editing of individual days within a shift pattern for quick changes, creation of custom shift patterns with multiple finishing times within a single pattern, and building shift patterns from existing shift patterns were highlighted (Figure 4). In addition, setting up recurring shift patterns with flexible cycles for users with nonidentical weekly or monthly schedules and providing notifications to alert users of upcoming shifts were suggested as key improvements. A few quotes from the sessions include the following:

I want to be able to edit days in shift pattern, including rest days and specific shift types.

M4P5

Sometimes there is need to manually change just one shift within a recurring cycle.

M5P4

We have officers with varying finish times, one pattern doesn’t fit all. Update the design to accommodate flexible shift time.

M4P3

It would help if the system recommended finish times for late and night shifts based on past inputs which can help with notification.

M4P2

It’s hard to understand systems designed to manage shifts for thousands of staff with varying start and end times.

M1P1

### How Can Consistency and Appropriate Terminology Enhance the Understanding of the Shift Scheduling System?

**Figure 5:**
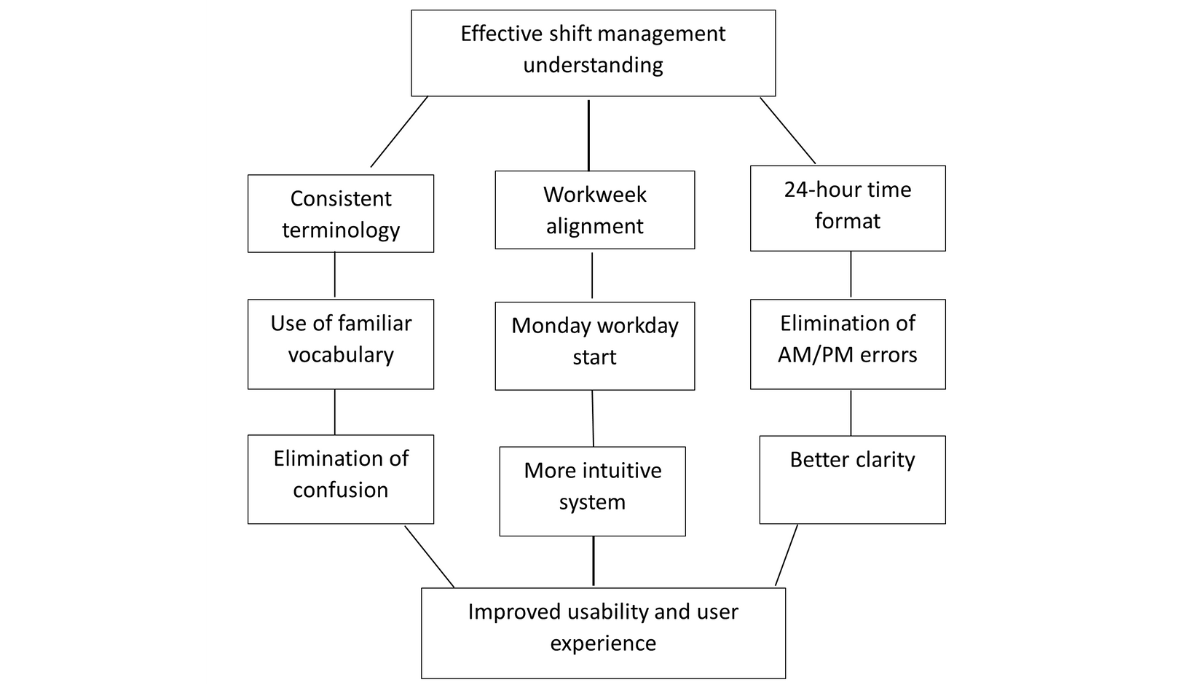
System understanding through terminology and time format consistency.

Participants indicated that replacing shift terms with more understandable and familiar vocabulary, as well as implementing consistent terminology throughout the system, significantly enhanced their ability to understand the shift management process. By reducing confusion and improving communication, participants felt more confident navigating the system. They also emphasized the importance of aligning shift patterns with the traditional workweek (starting on a Monday) and using a 24-hour time format, which further increased clarity and made it easier for them to manage their shifts effectively. Figure 5 shows how these changes can contribute to smoother operations and better communication across the system. A few quotes from the sessions include the following:

The system should use 24-hour time for consistency.

M2P5

We [police] usually begin our patterns on a Monday, so that should be the default.

M4P3

I would prefer more accurate labels instead of terms like “early,” they’re too vague, modify the term.

M2P1

### Results of the IM Workshop Session

The IM co-design workshops were conducted virtually via Microsoft Teams to promote participants’ convenience and were recorded for transcription and analysis. To facilitate idea generation, participants were guided using trigger questions specifically focused on improving the flexibility, usability, and effectiveness of shift scheduling systems to promote well-being support for police personnel. Participants’ ideas were recorded individually during the idea writing phase, and ideas were subsequently refined and discussed as a group and clarified collectively. One of the workshops was conducted on-site with members of the operational shift scheduling unit for policing departments.

Prioritization of ideas followed through nominal group techniques, allowing for categorization and construction of interpretive structural modeling.

The following trigger questions were used to prompt idea generation: (1) what enhancements can be made to improve the flexibility and customization of shift patterns? (2) How can shift management tools better accommodate personal and operational scheduling needs? (3) What features would improve communication and understanding of shift schedules?

Table 3 shows the ideas and participants’ responses generated using the trigger questions listed in Table 1. Table 5 shows that participants highlighted effective and flexible shift scheduling as an important requirement for a system capable of supporting the well-being of police personnel. The IM sessions also identified customization of schedules, shift monitoring, and schedule visualization as being crucial to the shift management system.

**Table 5:** Categorization of user-generated ideas into thematic groups.

Category	Ideas
Effective and flexible shift scheduling	1, 2, 7, 8, and 14
Customization of shift schedule	4, 5, 6, and 9
Shift monitoring and visualization	3 and 10
Consistency and terminology	11, 12, and 13
Pattern reusability	15 and 16

The nominal technique was used to allow participants to prioritize the top 5 requirements generated from the ideation phase, as shown in Table 5. The participants ranked the ideas from 1 (most important) to 5 (least important). This revealed that enabling bulk creation of shift schedules, creating reusable shift patterns, combining several shift types into a pattern, setting up recurring shift patterns with flexible cycles, and allowing for manual editing of daily shifts were ranked the highest among the participants.

According to Table 4, ideas 1, 6, 7, 9, and 16 were ranked as the top 5 requirements based on the total score.

**Figure 6:**
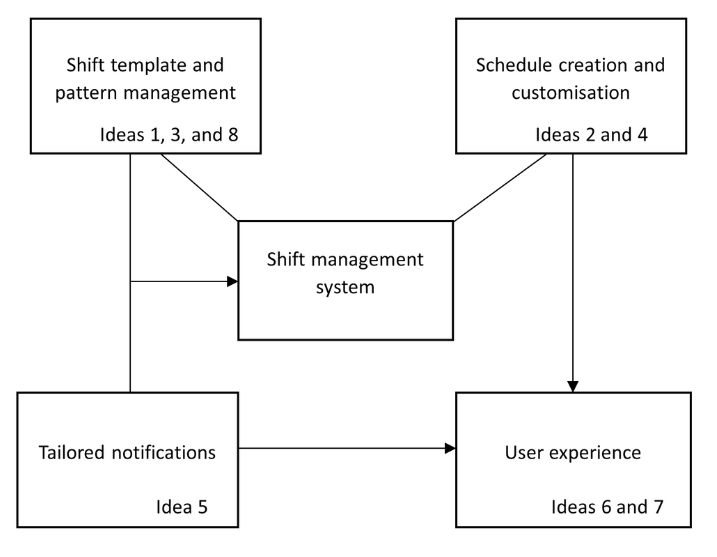
Interpretive structural model of prioritized system features.

On the basis of the prioritised ideas presented in Table 4, objective statements were formulated, that can be used to develop defined system requirements. These statements are outlined in the section below and were used to construct an interpretive structural model illustrated in Figure 6. The figure shows the categorization of similar objective statements into boxes, and the logical relationships between them.

### Interpretive Structural Modeling

Following prioritization, the top ideas were transformed into objective statements suitable for interpretive structural modeling (Figure 6). These statements represented the system requirements necessary to address user-identified needs: (1) enable reusable shift pattern templates with recurring cycle support to reduce manual effort and accommodate routine schedules; (2) allow for the bulk creation and deployment of shift schedules for efficiency across teams or departments; (3) support complex shift patterns by enabling the combination of multiple shift types within a single cycle; (4) allow for customization and manual editing of individual shift days to accommodate personal needs and real-time changes; (5) integrate notification features to remind users of upcoming shifts or updates; (6) use intuitive, standardized terminology to enhance user understanding and minimize confusion; (7) incorporate visual tools to monitor shift distribution, balance workloads, and identify scheduling issues; and (8) allow for the merging of rotating schedules to enable flexible, continuous coverage models.

### Prototype Design

#### First Prototype Iteration

**Figure 7:**
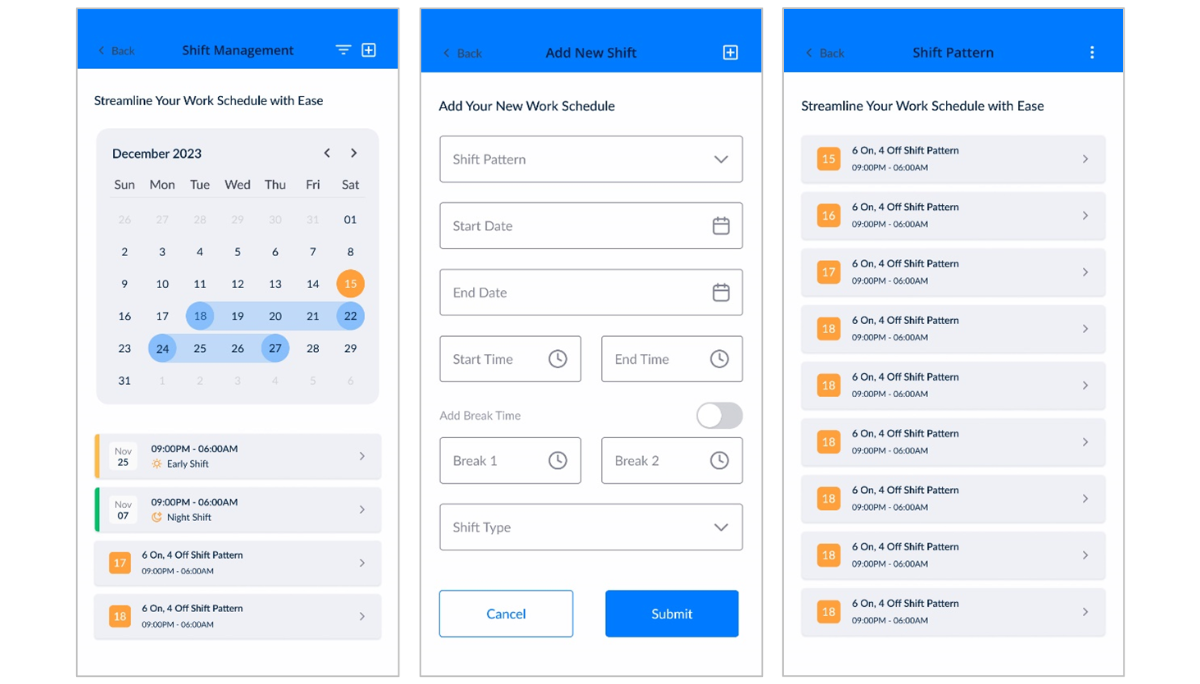
First iteration of the shift management system.

The prototype of the shift management system revealed several limitations that showed its ineffectiveness. One of the major issues was the lack of proper visualization, which made it difficult for users to identify trends and patterns in the various types of shifts (Figure 7A). This absence of clear, visual representation made it difficult to understand workload distribution and detect when a user is likely to be stressed from shift workload. In addition, the prototype did not account for varying finishing times of shifts, which is crucial for accommodating the diverse personal and professional schedules of police workers. Another significant shortcoming was the inability to merge multiple rotating shift pattern (Figure 7B), an important feature for efficiently managing schedules with complex, overlapping work times. The process of adding new shifts also involved too many steps, making the system time-consuming to use (Figure 7C).

#### Second Prototype Iteration: Refined Interface of the Shift Management System

**Figure 8:**
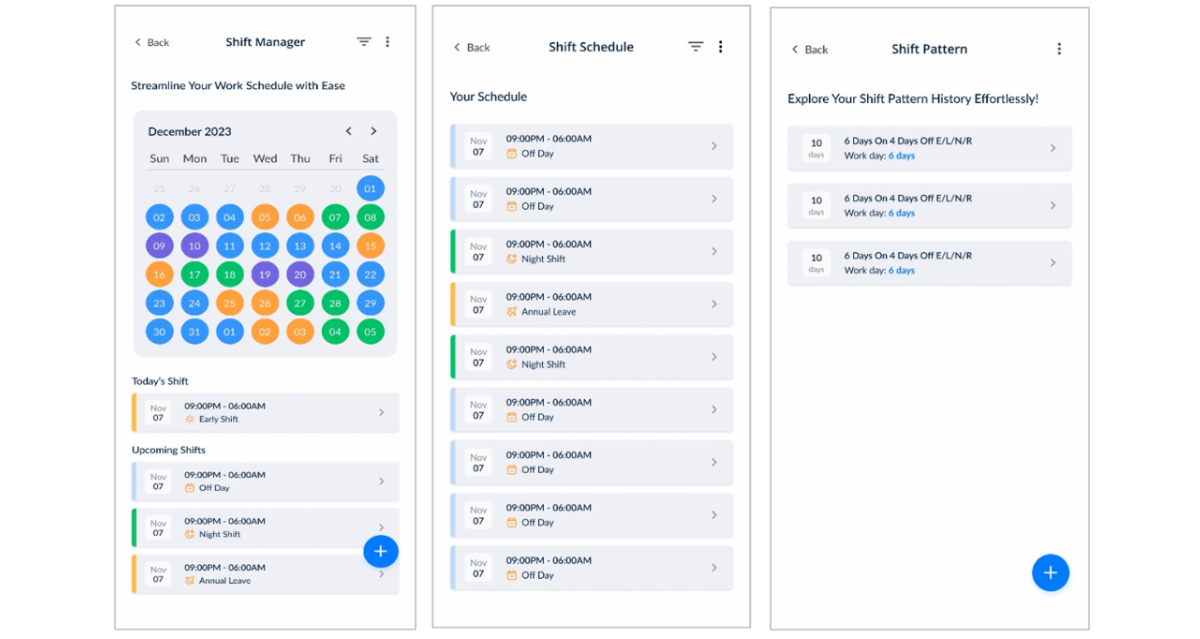
Refined iteration of the shift management system.

The second iteration of the shift management system was developed to address the issues identified in the initial prototype. One of the key improvements of this iteration was enhanced visualisation, which allowed users to more clearly identify trends in shift types and workload distribution, thereby supporting better decision-making (Figures 8A, 8B, and 8C).

**Figure 9:**
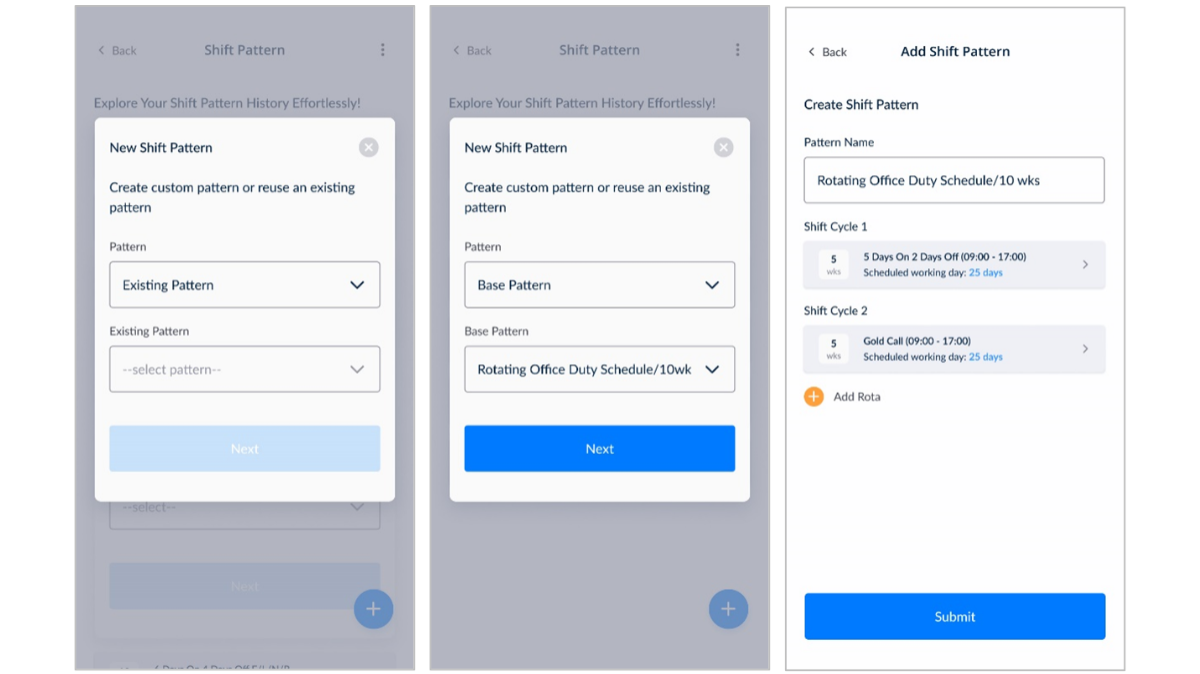
Base shift pattern reusability features.

The system introduced the pre-population of base shift patterns, which allowed users to reuse and modify existing patterns, significantly reducing the time and effort required to create new schedules. This feature, combined with the ability to adapt and modify previously created patterns to generate new ones provided additional flexibility and efficiency for users managing dynamic shift schedules (Figures 9A and 9B). The system also introduced functionality to merge multiple rotating shift schedules, which helped to streamline the management of complex team schedules (Figure 9C).

**Figure 10:**
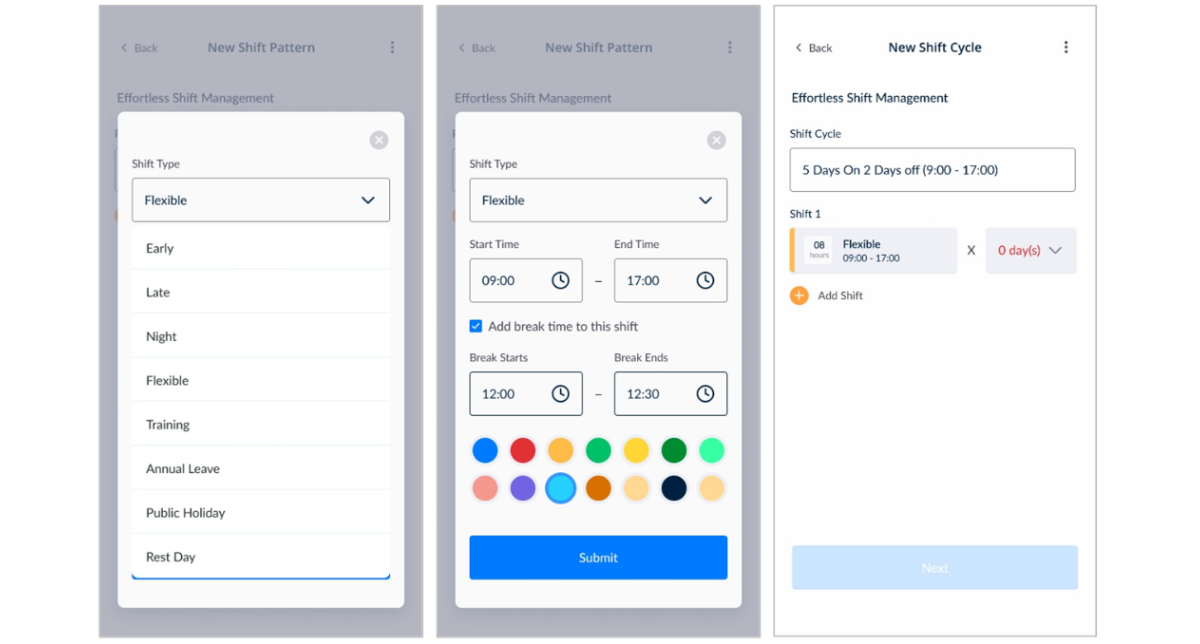
Shift type variability and finishing time customization.

Further enhancements included simplifying the process of adding new shifts, thereby reducing the number of steps and improving user-friendliness (Figure 10A). In addition, the system was updated to accommodate varying shift end times, providing more flexibility and customisation options for users with nonstandard or extended work hours (Figures 10B and 10C).

**Figure 11:**
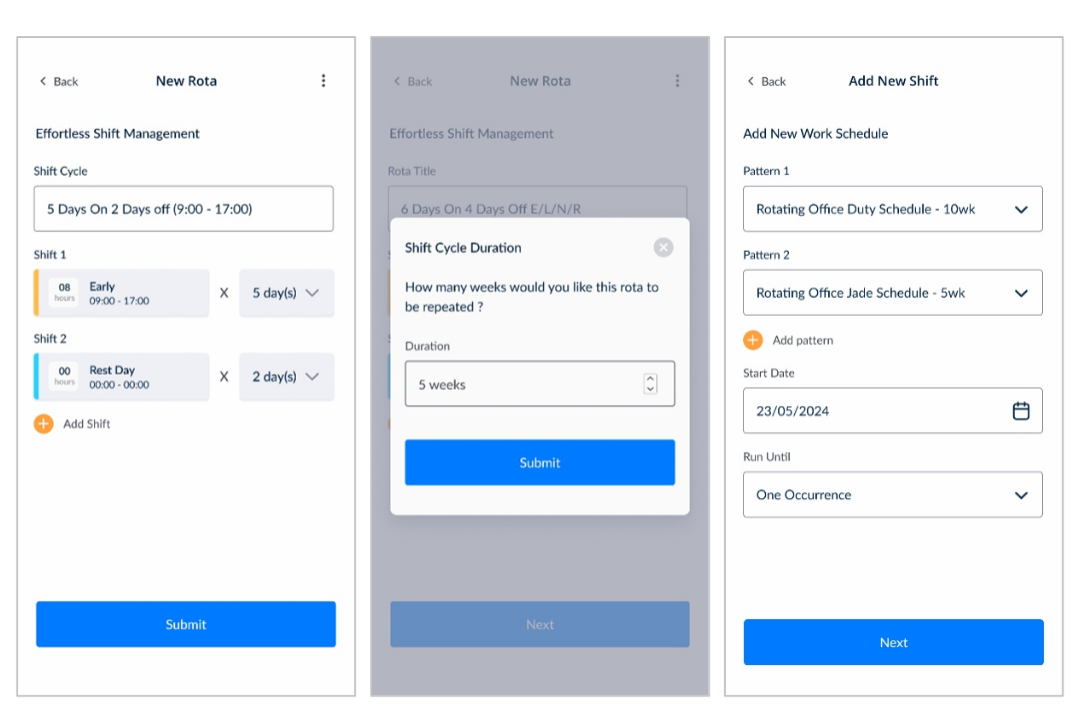
Recurring shift pattern design.

Finally, the iteration introduced consistent vocabulary and terminology to improve communication and reduce confusion. *Shift* was defined as a single working period (individual shift), *shift cycle* as a combination of shift and nonshift days over a working week, and *shift pattern* as one or more shift cycles distributed across a specified number of weeks. This standardisation in terminology helped users better understand and navigate the system (Figures 11A, 11B, and 11C).

Table 6 presents the functionality difference between the first and second iteration. The enhancements made the second iteration effective and user-friendly for the shift management system.

**Table 6:** Comparison of first and second iteration features of the shift management system.

Feature	First iteration	Second iteration
Shift scheduling	Basic, single shift type allocation	Bulk shift creation
Shift time customization	Fixed times	Daily time customization
Merging rotating schedules	Absent	Merging of multiple rotating schedules
Shift visualization	Limited and not color coded	Color-coded visualization
Notification	Absent	Notification for upcoming shifts added
Terminology	Unclear	Standardized terminology (“shift,” “shift cycle,” and “shift pattern”)
Preconfigured templates	Absent	Prepopulated base shift patterns and reusability of existing patterns
Recurring shift cycles	Absent	Recurring shift cycles with flexible cycle setup
Multiple finishing times	Absent	Supported within a single shift pattern
Shift creation process	Complex and requiring many steps	Simplified shift creation process
Time format	12-h format	24-h format
Start of shift patterns	Random start days	Shift pattern start aligned with Monday (traditional workweek)

In summary, the co-design process led to a substantial evolution of the prototype, resulting in a more refined and operationally aligned shift management system. The second iteration incorporated several key improvements that directly addressed the complex scheduling needs of police personnel. The iterated design included the introduction of bulk shift creation, flexible recurring shift cycles, merging of multiple rotating schedules, and daily shift time customization. The system also adopted a 24-hour time format, aligned shift pattern start dates to Mondays to reflect the traditional workweek, and introduced color-coded visualization and standardized terminology to enhance usability and clarity.

In addition, the integration of shift notifications and the reuse of preconfigured base patterns further increased the tool’s practicality and responsiveness to operational needs. The resulting prototype demonstrated a feasible and user-informed approach to digital shift management capable of supporting broader well-being goals for police personnel when integrated within a tailored DHI.

## Discussion

### Principal Findings

This study co-designed a digital shift management system tailored to the unique needs of UK police officers through 6 IM-facilitated co-design workshops. Key system features identified included flexible shift scheduling, visualization tools, preconfigured templates, daily time customization, and support for recurring patterns and last-minute changes. The resulting prototype reflected improved usability and operational alignment based on user feedback. This study highlights the value of UCD and participatory methods in enhancing the relevance and acceptance of digital tools. These findings highlight the importance of integrating shift management tools with digital health platforms. This integration would enable personalized well-being recommendations that support adequate hydration, nutrition, and physical activity; proper sleep quality; and sustainable work-life balance based on individual shift patterns.

The ideation results from the cocreation sessions revealed several key features that are important in developing an effective shift management system aimed at addressing the stress and workload challenges faced by police officers. These findings were generated from discussions with police workers and other stakeholders, providing experiences, scenarios, and insights into the demands of managing complex shift schedules.

Primary concerns identified included the lack of shift management features in digital health and well-being applications and the lack of flexibility in current shift management systems. These issues are particularly challenging for unconventional working hours and in handling last-minute changes such as extended hours or sudden shift adjustments, which significantly contribute to stress and work-life imbalance among police workers [3,16]. Functionality such as the bulk creation of shift schedules and flexible modification options emerged as critical for ensuring efficient management of large or block shift schedules and allowing for quick adjustments to accommodate personal or professional obligations [20]. Participants further stressed the need for daily shift time customization, which enables officers to balance their commitments with professional responsibilities. In addition, the manual editing of individual days within a shift pattern allowed for rapid adjustments without the need to recreate the entire schedule, thereby minimizing scheduling-related stress [49].

The ability to monitor shift types through visualization and the option to merge rotating schedules were also identified as important features. Regarding shift pattern design, participants recommended that shift patterns contain a combination of several shift types and that shift patterns with flexible cycles be offered. This would help manage the natural complexity associated with shift work and reduce disruptions that contribute to stress. Participants also highlighted the importance of the system being intuitive and user-friendly [70,76]. This includes using understandable and familiar vocabulary such as *shift*, *shift cycle*, and *shift pattern* and ensuring that shift patterns begin on a Monday to align with traditional workweeks. Such improvements would make the system more accessible and reduce the cognitive load required to interpret shift schedules. Furthermore, using a 24-hour time format across the system was suggested to ensure clarity and consistency in shift management [3].

Finally, the cocreation sessions pointed to the benefit of prepopulating the system with common base shift patterns that can be reused and modified, significantly reducing the time and effort required for creating new shift schedules. The ability to create schedules with multiple finishing times would accommodate the varying shift needs of officers. In addition, notifications to alert police personnel of upcoming shifts would help reduce last-minute scheduling surprises and associated stress, ensuring better communication and preparedness. Integrating planned or scheduled work breaks within each shift would be beneficial to police personnel in reducing fatigue, improving their focus, and promoting their overall health and well-being, thereby improving performance and job satisfaction [8,32,77,78].

Overall, these findings highlight the need for a shift management system that prioritizes flexibility, clarity, and real-time adaptability to accommodate the unpredictable nature of police work. The system must allow for the seamless input and modification of shift schedules, enabling officers to manage surprise schedule changes and long hours easily. This capability is essential to reduce the stress caused by the complex and demanding nature of shift work. The shift management system should both address operational challenges and play a critical role in enhancing officer well-being by providing real-time notifications and recommendations that reflect these sudden adjustments [77].

### Evaluating the Relationship With Previous Findings

The findings of this study align with the lack of shift schedule personalization in existing health and well-being interventions for emergency workers such as police officers [3]. Studies on existing mHealth tools have revealed that, while apps such as Healthier Outcomes at Work [39], the #SWPMoveMore Challenge intervention [41], Headspace [37], Sleepfit [38], and Healthy Minds Innovation [40] address various aspects of health, they are not tailored to the specific needs of shift workers. These apps offer crucial interventions, such as workplace stress management, mindfulness, sleep health, and physical activity support, but lack integration with real-time shift schedules, thus making them limited in meeting the unique demands of populations such as police officers and emergency workers who experience unpredictable and irregular work patterns.

Compared with existing literature on digital stress interventions, the shift management tool codeveloped in this study shows alignment with the findings of Weerasekara and Smedberg [2], who emphasize user preferences for personalized and flexible digital solutions. By incorporating UCD principles [68] and IM [58], this study contributes to research advocating for participatory design [60] as a means of increasing the acceptability and usability of mHealth tools. The emphasis on adaptive scheduling, modular functionality, and simplified workflows reflects existing best practices for improving digital engagement and reducing occupational stress [79], fatigue [80], and depression in the workplace [81] through digital platforms [46,49]. Notably, the inclusion of domain experts in shift coordination, digital health, and HCI enhanced the quality of design decisions and ensured alignment with real-world operational constraints.

### Limitations

This study has several limitations. First, the relatively small participant sample size may limit the generalizability of the findings beyond the immediate police force context. Second, despite the diversity of professional roles represented in the co-design workshops, broader input from frontline personnel across multiple forces would have further enhanced the design applicability. Third, no long-term deployment or efficacy testing was conducted within this study phase, meaning that impacts on stress or job performance remain unmeasured. Finally, the IM workshops, though highly structured and effective for consensus building, required substantial facilitation and planning efforts that may not be feasible in all contexts.

### Conclusions

This study co-designed a shift management system whose effectiveness and impact can be more thoroughly evaluated and used when integrated into a broader health and well-being application tailored to the needs of shift workers. This study concluded that developers and designers should create DHIs that are tailored to the needs of professions with irregular shift schedules to ensure real-time adaptability and personalization. Organizations specifically within emergency services should encourage the adoption of health and well-being solutions that promote employees’ health, job satisfaction, and quality of life. Finally, more collaborations among health experts, frontline personnel, and technology experts should be encouraged to ensure that digital health solutions are relevant and sustainable in addressing the challenges associated with shift work.

## Appendix

Multimedia Appendix 1 Supplementary materials.

## Abbreviations

DHI: digital health intervention
HCI: human-computer interaction
IM: interactive management
mHealth: mobile health
TA: thematic analysis
UCD: user-centered design
