# Supplementary material for: Supporting Police Well-Being Through an Adaptive Shift Management System: Co-Design Study
Source: JMIR Form Res. 2025 Aug 28;9:e69986. doi: 10.2196/69986 (PMC12426568; doi:10.2196/69986)
Supplement: Multimedia Appendix 1 [file formative_v9i1e69986_app1.docx]

**Code Development from Co-creation Session**

| **S/N** | **Quotes** | **Participants** | **Keywords** | **Themes** |
| --- | --- | --- | --- | --- |
| 1 | Current system has limited flexibility and inconsistent scheduling. | M1P3 | flexibility, inconsistency, scheduling, system | Challenges in shift management |
| 2 | Flexible or predictable scheduling system would help maintain a better work-life balance. | M1P4 | flexibility, predictability, scheduling, work-life balance | Impact on work life balance |
| 3 | Poorly designed shift patterns harm personal life and work performance. | M1P5 | poorly designed, shift patterns, personal life, work performance | Challenges in shift management |
| 4 | Struggle with the complexity of software made for large teams, especially when everyone has different schedules. | M1P2 | complexity, software, large teams, schedules | Challenges in shift management |
| 5 | It's hard to understand systems designed to manage shifts for thousands of staff with varying start and end times. | M1P1 | systems, shift patterns, employees, start times, end times | Challenges in shift management |
| 6 | I know there will be concern about what it means to adopt a completely new shift management system. | M1P5 | adoption, new concern on new system, shift management | User adaptability and training |
| 7 | I think it would help to have a model or table that links user personas with shift patterns | M2P1 | model, mapping, personas, shift patterns | Persona development |
| 8 | We should create 5 to 10 fictional personas based on demographic data and real shift schedules | M2P5 | personas, demographic, shift schedules, fictional | Persona development |
| 9 | I would like to see a list of example characters with typical shift patterns to guide system use | M2P3 | example characters, shift patterns, guide, use | Persona development |
| 10 | Using terms like ‘day’, ‘late’, and ‘night’ would be simpler than listing exact times | M2P4 | descriptors, day, late, night, simplify | Simplified shift terminology |
| 11 | I would prefer more accurate labels instead of terms like ‘early’, they’re too vague | M2P1 | terminology, accuracy, labels, shift times | Simplified shift terminology |
| 12 | The system really should use 24-hour time for consistency | M2P5 | 24-hour, time format, consistency | Consistence use of terms and Time format |
| 13 | A tool that can manage different shift types in a single interface | M2P2 | shift types, manage, single interface, tool | Schedule creation and management |
| 14 | Rotating shifts are difficult to manage, especially when we have to combine multiple patterns. | M3P1 | rotating shifts, management, combination, patterns | Challenges in shift management |
| 15 | We often start with Gold or Silver base patterns and tweak them to create new schedules. | M3P1 | base patterns, Gold, Silver, schedules | Custom and pre-set shift pattern |
| 16 | It would really help to have templates specific to different departments pre-loaded into the system. | M3P1 | templates, departments, pre-loaded | Custom and pre-set shift pattern |
| 17 | I want the option to manually create shift patterns, not just rely on templates. | M3P3 | manual creation, shift patterns, templates | Custom and pre-set shift pattern |
| 18 | Sometimes I need to build a custom shift pattern based on an existing one. | M3P1 | custom pattern, base template, build | Custom and pre-set shift pattern |
| 19 | I reuse the same base pattern frequently, and I’d like the system to support that. | M3P1 | reuse, base pattern, support | Custom and pre-set shift patterns. |
| 20 | The flexibility to either reuse an old pattern or create a new one would be ideal. | M3P2 | reuse, existing patterns, new patterns, flexibility | Custom and pre-set shift patterns. |
| 21 | If I need to change something mid-week, I basically have to start over. | M4P2 | changes, mid-week, restart | Usability |
| 22 | I want to be able to edit days in shift pattern, including rest days and specific shift types. | M4P5 | edit, rest days, shift types, pattern | Usability |
| 23 | We should simplify things by using cycles instead of overly complex shift patterns. | M4P1 | simplify, cycles, complexity | Simplification of features |
| 24 | ‘Day’, ‘late’, and ‘night’ make more sense than specific start and end times. | M4P4 | descriptors, simplify, shift types | Simplified shift terminology |
| 25 | I need to change shift times even when a pattern is already active or in use. | M4P3 | edit, shift times, active pattern | Flexible shift times, simplification of features |
| 26 | Interactive visual tools would make it easier for me to track progress over time. | M4P1 | visualisation, tools, tracking, progress | Interactive features |
| 27 | Users will benefit from being able to see trends that show recurring scheduling issues. | M4P5 | trends, scheduling issues, tracking | Interactive features |
| 28 | It would help if the system recommended finish times for late and night shifts based on past inputs which can help with notification | M4P2 | default finish times, night shift, notifications | Flexible shift time, shift notification |
| 29 | Users should be able to set different shift times for different days within the same pattern. | M4P4 | flexible, shift times, daily pattern | Usability, Flexible Shift times, Shift customisation |
| 30 | We have officers with varying finish times, one pattern doesn’t fit all. Update the design to accommodate flexible shift times. | M4P3 | bespoke, officers, finish times | Flexible shift time, custom shift pattern |
| 31 | Entering work patterns takes too long. It should be simpler. | M4P5 | work patterns, input, simplicity | Custom shift pattern, usability |
| 32 | I need to include rest days as part of the shift definition. | M4P1 | rest days, selection, shift types | Shift creation and management |
| 33 | It would be nice if patterns could start on a typical workday. | M4P2 | workday, pattern start | Shift pattern, start date |
| 34 | We (police) usually begin our patterns on a Monday, so that should be the default. | M4P3 | Monday, start, default | Shift Pattern, start Date |
| 35 | The pattern should always start at the beginning of the work week, it avoids confusion. | M4P4 | work week, beginning, pattern | Shift pattern, start date |
| 36 | If it doesn’t start on Monday, it throws everyone off. | M4P5 | confusion, start date, Monday | Shift Pattern, start date |
| 38 | Some of the shift labels don’t match what we use day-to-day, which makes it confusing. | M4P2 | shift vocabulary, match, usage | Simplified shift terminology |
| 39 | Using colour to identify different shift types would make the calendar easier to read. | M4P1 | colour-coded, shift types, identification | Interactive features |
| 40 | I want to see my weekly schedule all in one view, not just one day at a time. | M4P3 | weekly schedule, overview, display | Usability |
| 41 | The layout feels cluttered when there are too many shifts showing at once. | M4P4 | layout, cluttered, visual overload | Usability |
| 42 | Manually adding shifts takes too long, we need a quicker way to do it. | M4P5 | manual entry, time-consuming, shift creation | Usability |
| 43 | It would be nice to merge two patterns together and use them as one ongoing schedule. | M5P2 | merge, shift patterns, continuous | Shift creation and management |
| 44 | If I could just change one day without messing up the whole pattern, that would be great. | M5P1 | edit, single shift, flexibility | Usability |
| 45 | I would prefer different colours for sick leave, annual leave, and normal shifts to keep things clear. | M5P3 | colours, leave types, clarity | Interactive features |
| 46 | I would appreciate reminders to take breaks when I’ve been doing back-to-back shifts. | M5P5 | reminders, breaks, consecutive shifts | User adaptability |
| 47 | Sometimes there is need to manually change just one shift within a recurring cycle | M5P4 | manual edit, recurring cycle, day shift | Usability |
| 48 | I want to input my entire schedule in one go like a bulk pattern, not break it into separate parts. | M5P2 | bulk input, full schedule, efficiency | Shift creation and management, usability |
| 49 | I would like to combine multiple work schedules into one clean view. | M5P1 | multiple schedules, combination, unified view | Simplification of features |
| 50 | I would like the option to customise notifications and see tips when I’m new to the system. | M6P4 | customise, notifications, tips | Shift notification, demonstration, and validation |
| 51 | There should be a simple way to describe different shift options and how we use them. | M6P1 | shift options, description, interaction | User adaptability and training, demonstration and validation, interactive features |
| 52 | The system should suggest breaks when we’ve worked several heavy shifts in a row. | M6P5 | suggest, breaks, heavy shifts | User adaptability |
| 53 | How do we make sure people get enough training and guidance when rolling this out? | M6P3 | training, guidance, rollout | User adaptability and training |
| 54 | The system should give me clear instructions, like how to start a pattern on a certain day and repeat it. | M6P2 | shift options, description, interaction | Usability |
| 55 | It would be helpful to get tips and alerts when using the system for the first time. | M6P5 | notifications, tips, first-time users | Shift notification, demonstration, and validation |
| 56 | The system should be able to warn me if I’m scheduled to work too many night shifts in a row. | M6P4 | warnings, night shifts, scheduling | User notification |
| 57 | I might need training or support to use this system confidently. | M6P1 | support, training, confidence | User adaptability and training |
